# Nanoparticles for the Induction of Antigen-Specific Immunological Tolerance

**DOI:** 10.3389/fimmu.2018.00230

**Published:** 2018-02-20

**Authors:** Takashi Kei Kishimoto, Roberto A. Maldonado

**Affiliations:** ^1^Selecta Biosciences Inc., Watertown, MA, United States

**Keywords:** nanoparticles, immunological tolerance, rapamycin, tolerogenic dendritic cells, regulatory T cells

## Abstract

Antigen-specific immune tolerance has been a long-standing goal for immunotherapy for the treatment of autoimmune diseases and allergies and for the prevention of allograft rejection and anti-drug antibodies directed against biologic therapies. Nanoparticles have emerged as powerful tools to initiate and modulate immune responses due to their inherent capacity to target antigen-presenting cells (APCs) and deliver coordinated signals that can elicit an antigen-specific immune response. A wide range of strategies have been described to create tolerogenic nanoparticles (tNPs) that fall into three broad categories. One strategy includes tNPs that provide antigen alone to harness natural tolerogenic processes and environments, such as presentation of antigen in the absence of costimulatory signals, oral tolerance, the tolerogenic environment of the liver, and apoptotic cell death. A second strategy includes tNPs that carry antigen and simultaneously target tolerogenic receptors, such as pro-tolerogenic cytokine receptors, aryl hydrocarbon receptor, FAS receptor, and the CD22 inhibitory receptor. A third strategy includes tNPs that carry a payload of tolerogenic pharmacological agents that can “lock” APCs into a developmental or metabolic state that favors tolerogenic presentation of antigens. These diverse strategies have led to the development of tNPs that are capable of inducing antigen-specific immunological tolerance, not just immunosuppression, in animal models. These novel tNP technologies herald a promising approach to specifically prevent and treat unwanted immune reactions in humans. The first tNP, SEL-212, a biodegradable synthetic vaccine particle encapsulating rapamycin, has reached the clinic and is currently in Phase 2 clinical trials.

## Introduction

The central function of the immune system is the maintenance of immunological tolerance to self-components and innocuous exogenous antigens while eliminating malignant cells and dangerous pathogens. Immunological tolerance, defined as the absence of immunity to an antigen even in the presence of otherwise immunogenic stimuli, is achieved through a combination of processes that lead to the elimination or inactivation of immune cells specific for the antigen and the development of regulatory T cells (Tregs). The first and most impactful selection process, called central tolerance, eliminates lymphocytes recognizing self-antigens or leads to the differentiation of natural Tregs in the thymus. Autoreactive cells can escape this process and survive to join the repertoire of mature circulating lymphocytes. This pool of potentially dangerous cells can be further tolerized by encountering their cognate antigen in absence of immunogenic signals leading to anergy or the induction of adaptive Tregs and the establishment of peripheral tolerance (1). For example, autoreactive lymphocytes specific for components of the nervous system can be identified in the circulation of animals and humans (2, 3). These cells remain dormant and checked by regulatory T and B cells. Similarly, most “foreign” gut-associated antigens (microbial or dietary) are well tolerated and do not trigger pathogenic immune responses. However, in presence of strong and persistent stimuli, lymphocytes specific for these antigens can break tolerance and launch attacks against self-components and innocuous antigens triggering disorders such as autoimmune diseases and food allergies, respectively.

**Figure 1:**
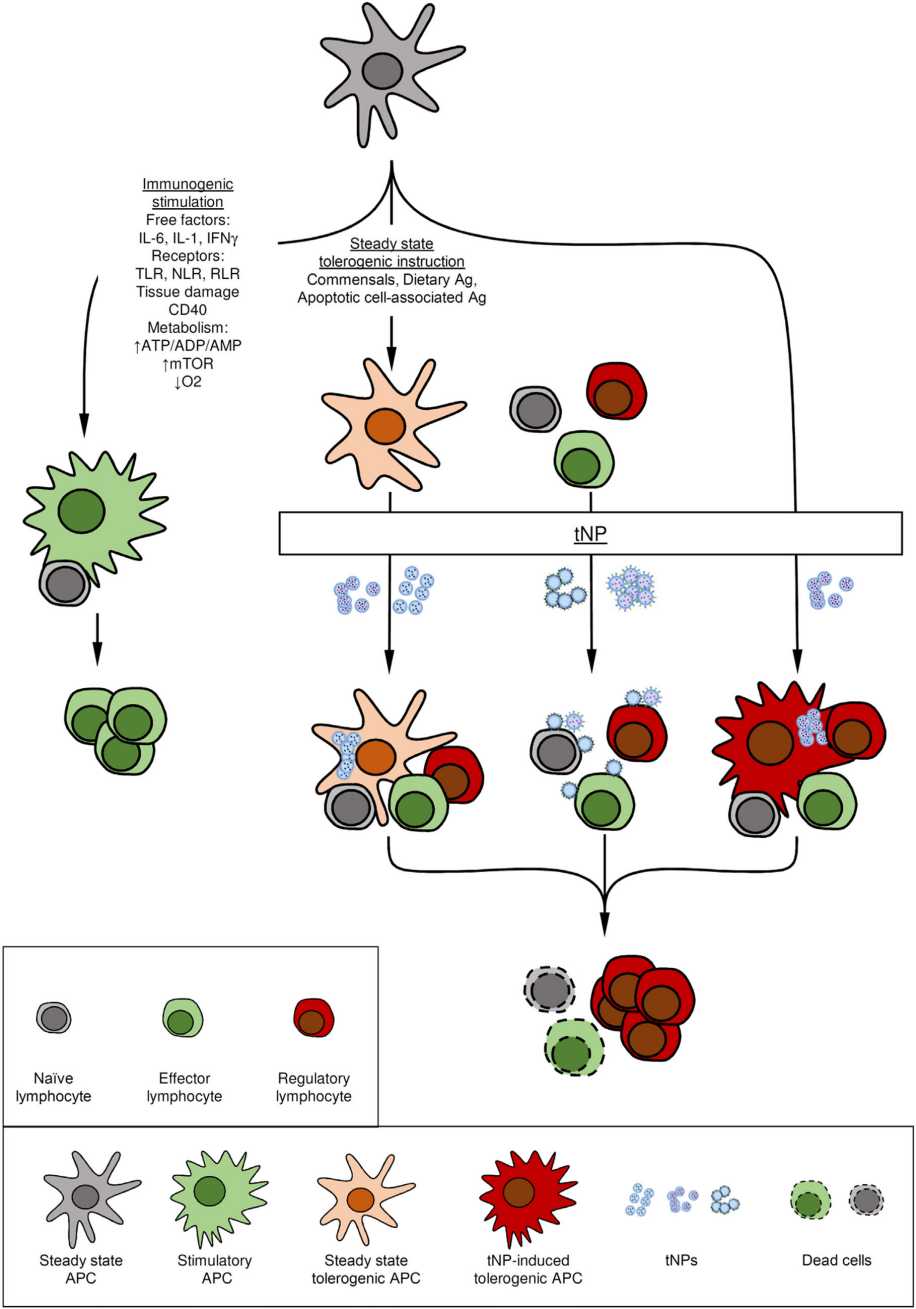
Mode of action of tolerogenic nanoparticles (tNPs). Antigen-presenting cells (APCs) play a major function in the immune system by integrating cues from the environment to promote immunity or tolerance. (A) Immunogenic stimuli such as cytokines, microbial components recognized by toll-like receptors (TLRs), NOD-like receptors (NLRs), RIG-I-like receptor (RLR), and changes in the metabolic state of the tissue can activate APCs to induce immunity. (B) At steady-state immature APCs that capture self and innocuous antigens, such as those from commensal bacteria, present antigen in the absence of costimulation to induce or maintain tolerance. (C) Some tolerogenic NPs harness these natural tolerogenic processes by targeting tolerogenic delivery routes (oral tolerance), tolerogenic environments (liver), or mimicking apoptotic cells. (D) Other tNPs actively promote immune tolerance by employing pharmacological agents to induce tolerogenic dendritic cells. (E) Lymphocytes can also be targeted directly by tNPs that engage antigen-specific receptors in absence of costimulation or by targeting tolerogenic receptors. In all cases, tolerance is mediated by the preventing the activation of or eliminating antigen-specific cells both (naïve or effector) and/or the expansion of regulatory lymphocytes.

Antigen-presenting cells (APCs), such as dendritic cells (DCs), are at the crossroads of immunity and tolerance (Figure 1). APCs sample and process antigens in the context of multiple complex cues from their environment. The pivotal signals allowing APCs to instruct lymphocytes to acquire the expression of costimulatory molecules and support the development of immunity have been categorized as “danger signals.” Such signals include pathogen-associated molecular patterns (4), damage-associated molecular patterns (5), changes in the tissue metabolic state (6), inflammatory cytokines (7), and costimulatory-molecule ligands (8). Stimulation of APCs triggers a “maturation” program that includes activation of the NF kappa B (NF-κB) and mammalian target of rapamycin (mTOR) pathways and leads to metabolic changes and upregulation of costimulatory molecules, such as such as CD80, CD86, and CD40, and production of pro-inflammatory cytokines (9–11). By contrast, antigen presentation in the absence of such costimulatory signals results in anergy and tolerance (12, 13). APCs capable of tolerance induction include macrophages, B cells and DCs (14–17). Animals lacking DCs have a general failure in the establishment of self-tolerance, resulting in autoimmune conditions (18–22). Whether an immature or steady-state phenotype is required for DCs to induce tolerance is still a matter of debate. Recently the notion that tolerance is established by DCs that undergo incomplete maturation has been challenged by findings that tolerogenic DCs require transcriptional and epigenetic programs distinct from both steady-state (immature) and activated (mature) DCs (14, 19, 22–24). Furthermore, there is conflicting evidence about the phenotypic characteristics that define tolerogenic DCs induced by immunomodulatory drugs. For example, induction of tolerogenic capacities by treating DCs *in vitro* or *in vivo* with free or encapsulated rapamycin results in induced tolerogenic DCs (itDCs) of different phenotypes and maturation characteristics (e.g., expression of MHC-CLII and costimulatory molecules) (14, 25–30).

Regardless of the specifics of their phenotype, APCs constitute an ideal target to manipulate immune responses (Figure 1). Nanoparticles have unique properties that make them well suited to target APCs and deliver instructions that can modulate the nature of an antigen-specific immune response *in vivo* (31–35).

## Why Nanoparticles?

The immune system has evolved to capture and interrogate virus-like (nanosized) particles (36, 37). Such nanoparticulates are filtered out and accumulate in lymphoid organs, such lymph nodes and the spleen, and the liver. This scavenger task is performed by APCs that are adept at phagocytosing and eliminating debris in the extracellular environment. Synthetic nanoparticles of a wide array of materials in the range of 50 nm to 1 µm of size are readily phagocytosed by APCs (31, 32, 36–38). The display of multimerized antigen on nanoparticles has been shown to be inherently immunogenic, similar to particulate or aggregated antigen (36, 37, 39). Encapsulation or conjugation of antigens (both peptides and entire proteins) can lead to their presentation as a multimerized complex that has the potential to directly engage and cross-link of B cell receptors (BCRs), resulting in the activation of humoral immunity. Indeed, many particle-based vaccines exploit these principles (encapsulation and multimeric display) to induce protective humoral immunity (38).

To engineer nanocarriers for the induction of tolerance, we and others have use materials and components that provide tolerogenic signaling to APCs or harness natural tolerogenic processes to override the inherently immunogenic nature of antigen-bearing nanocarriers. The usage of synthetic tolerogenic nanoparticles (tNPs) confers several important advantages compared with other strategies to induce tolerance (Table 1). Nanoparticles can employ a wide range of materials that can be optimized for various functions and can carry a diverse payload of antigens and immunomodulators to deliver coordinated messages to the immune system.

**Table 1:** Tolerogenic nanoparticle (tNP) composition, mechanism, and characteristics.

tNP composition	Mechanism	Characteristics	Reference
Peptide–major histocompatibility complex (MHC) complexes on metal-oxide NPs or peptide–MHC complexes plus anti-Fas ligand antibody	Antigen presentation w/o costimulation on synthetic antigen-presenting cell. Anti-FAS ligand antibody delivers apoptotic signal	Direct action on effector T cells, but requires complex manufacturing. Restricted to peptide antigens (antigen selection risk). Non-biodegradable	(40–42)
Protein or DNA-encoded antigen in poly(lactic-*co*-glycolic acid) (PLGA) or chitosan NPs	Oral tolerance	Ease of delivery *via* oral route. However, poor history of translation for oral tolerance	(43–45)
Peptides conjugated to polystyrene, PLGA, or poly(maleic anhydride-alt-1-octadecene) nanoparticles	Mimic apoptotic cells; target tolerogenic niche *via* MARCO+ macrophages in spleen or liver sinusoidal cells	Simple composition, but restricted to peptides and i.v. dosing. Potential to be stimulatory in inflammatory setting	(46–52)
Antigen encapsulated in liposomes containing phosphatidylserine (PS)	Mimic apoptotic cells TAM? Scavenger receptor-mediated uptake by macrophages	PS-binding scavenger receptors trigger TAM? receptors and tolerogenic response	(53–57)
NPs encapsulating tolerogenic cytokines and antigen	Anti-inflammatory cytokines create a tolerogenic microenvironment?	Complex manufacturing. Potential to create autoreactive immune response to endogenous cytokines	(58–60)
Liposomes presenting antigen and CD22 ligand	Induce antigen-specific B cell tolerance and deletion	Direct action on specific B cells. CD22 ligand is a complex sugar that is difficult to manufacture. Requires protein antigen	(61, 62)
Gold particles presenting peptide antigen and aryl hydrocarbon agonist	Trigger aryl hydrocarbon receptor (AHR) pathway	Utilizes an immunomodulator (AHR agonist) to lock in tolerogenic response. Restricted to peptides? Non-biodegradable	(63, 64)
Liposomes containing peptide antigen and antigen	Inhibit NF kappa B (NF-κB) pathway	Utilizes an immunomodulator (NF-κB inhibitor) to lock in tolerogenic response. Works with protein antigens and s.c. or i.v. route	(65)
Polylactic acid/PLGA NPs containing rapamycin + antigen (encapsulated or free)	Induce tolerogenic dendritic cells by inhibition of mammalian target of rapamycin pathway	Utilizes an immunomodulator (rapamycin) to lock in tolerogenic response. Works with both protein and peptide antigens and s.c. or i.v. route. Human proof of clinical activity demonstrated	(30, 66–72)

**Figure 2:**
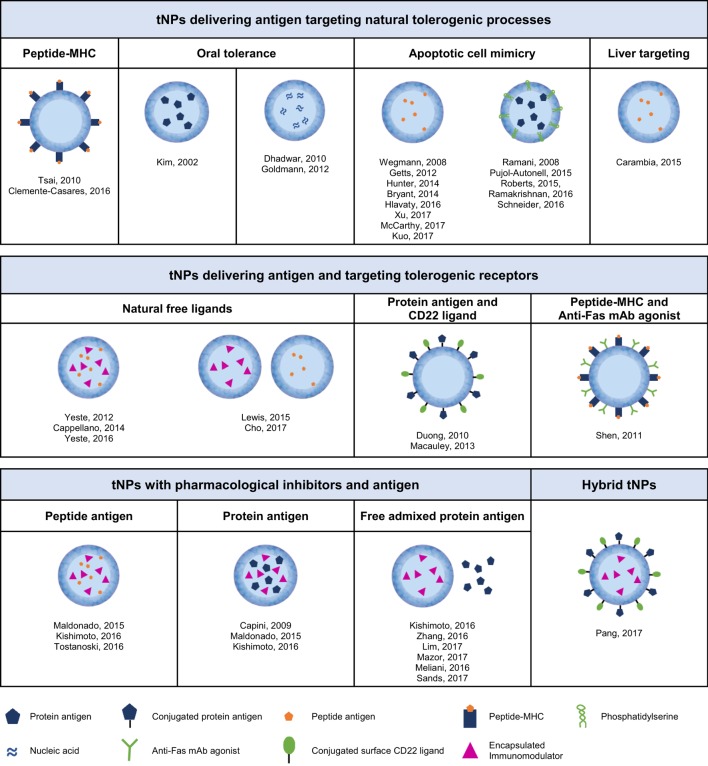
Different types of tolerogenic nanoparticles according to their content and mechanism of action.

This review will focus on nanoparticle approaches for the induction of antigen-specific immune tolerance. We define antigen-specific tolerance as the absence of immune response against an immunogenic target antigen, maintenance of tolerance after cessation of treatment, and retention of the ability to mount an immune response to an unrelated antigen. There have been three broad approaches to achieving antigen-specific immune tolerance with nanoparticles (Figure 2): (1) tNPs that provide antigen alone to harness natural tolerogenic processes or environments, (2) tNPs that provide antigen while targeting pro-tolerogenic receptors, and (3) tNPs that use pharmacological immunomodulators to force or “lock-in” a tolerogenic immune response against a target antigen. Nanoparticle delivery of immunomodulators, in the absence of a specific target antigen, for the treatment of autoimmune diseases and prevention of graft rejection is beyond the scope of this review, although it is notable that this approach has demonstrated durable disease modification in animal models (73–76). Similarly, nanoparticles that skew the immune response in an antigen-specific manner, such as Th1 polarizing nanoparticles for the treatment of Th2-mediated allergic diseases (77), are also not included in this review.

## tNP Properties

Key attributes of nanoparticles affecting their function include material composition, size and charge. Materials for tNP manufacturing are diverse, and the choice depends on the desired function and compatibility with the payload. Three broad categories of materials include metals, liposomes, and synthetic and natural polymers. Metal and metal-oxide nanoparticles have been used for theranostics with capacity to carry antigens, targeting ligands, and immunomodulators on the particle surface (40, 41, 63, 64). These particles are very stable but typically require conjugation of the payload which may limit the application to certain molecules. A key disadvantage is that metal particles are not biodegradable, and hence accumulation may become a safety issue. Liposomes have been used in the clinic to deliver small molecule drugs and nucleic acids. Liposomes can incorporate various different phospholipids to create a membrane bilayer. The addition of cholesterol can alter the membrane fluidity of the lipid bilayer, which enables clustering of surface molecules upon interaction with target cells (62). In addition, phosphatidylserine (PS) lipids can be incorporated to target scavenger receptors involved in the phagocytosis of apoptotic cells (53). Liposomes can be adapted to incorporate various payloads with different physicochemical properties that get released after the liposome fuses with a cell membrane or after degradation in endosomes. The manufacturing of liposomes through a low shear extrusion method allows for encapsulation of proteins while minimizing the risk of denaturation. Molecules can be conjugated to the surface of liposomes but also hydrophilic molecules are amenable for encapsulation within the aqueous core of liposomes, while hydrophobic molecules can intercalate into the membrane bilayer. The release kinetics of the payload may be difficult to fine tune with liposomes. Various both natural and synthetic polymers have been used for tNP manufacturing, including polylactic acid (PLA), poly(lactic-*co*-glycolic acid) (PLGA), polystyrene, acetylated dextran, poly-l-lysine, polyacrylamide, and chitosan. The use of biodegradable polymers is preferred for safety. Biodegradable PLGA/PLA polymers have an excellent safety profile and have been used in various approved drugs and medical devices. PLGA/PLA tNPs are formulated by an evaporation emulsion method. Unlike liposomes, PLA/PLGA nanoparticles are solid particles in which the payload is embedded within the matrix. Hydrophobic molecules, such as rapamycin, dexamethasone, and vitamin D3, can be dissolved together with the polymer in a solvent and then emulsified with an aqueous phase-containing surfactants such as polyvinyl alcohol and pluronic (57, 59). Proteins and hydrophilic molecules can be incorporated through a double emulsion process (78). The release rate of the payload can be fine tuned by altering the glycolic acid to lactic acid ratio in PLGA, by changing the size of the polymer, and the use of excipients. The release rate can be further modified by conjugating molecules to the polymers which enables self-assembly of nanoparticles with the target molecules displayed on the surface or embedded within the matrix of the particles, as desired (79). The high shear process used to create nanoparticles may result in partial denaturation of proteins. However, if the protein payload is an antigen, then denaturation is not an issue, as the antigen is targeted for degradation by APCs. Functional proteins can be attached to the surface of nanoparticles to preserve protein structure and function.

Nanoparticle surface charge has a strong influence on the immune responses. Typically, cationic nanoparticles lead to pro-inflammatory responses while anionic surfaces display reduced immunogenicity and longer circulation times. Anionic PLGA tNPs with zeta potential values in range of −40 to −70 mV have been shown to target a specific population of macrophages in the spleen expressing the scavenger receptor MARCO (48, 49, 51, 80, 81). In addition, many nanoparticle technologies use polyethylene glycol (PEG) to create a surface that is less prone to aggregation and opsonization by blood proteins, resulting in improved blood circulation properties (82, 83). Size can also affect circulation time and biodistribution. Particles smaller than 6 nm drain to the blood whereas particles larger than 9 nm tend to preferentially drain to lymphatics (84). Nanoparticles in the range of 20–100 nm have been shown to accumulate in the liver in liver sinusoidal endothelial cells (LSECs) or macrophages upon i.v. injection while after s.c. injections bigger particles have a tendency to become trapped in the extracellular environment and require active transport by phagocytes to reach the draining lymph nodes (85–88). Particles from 100 to 200 nm can traffic to both the spleen and liver when injected i.v. and circulate through the lymph after s.c. injections to be taken up by lymph sinus DCs that accumulate in draining lymph nodes (88, 89). Bigger particles from 200 nm to 5 µm accumulate mostly in the spleen when injected i.v. and require active cellular transport to reach lymph nodes when injected s.c. (50, 66).

Ultimately, the choice of materials comes down to optimizing compatibility with the desired payload and activity. Within each class of material, there is a considerable range of options that can be used to optimize nanoparticles for specific payloads (e.g., immunomodulators) and activity (e.g., release rates). For example, there are different types of metals that can be used, a range of lipids that impart different properties to liposomes, and different types of polymer chemistries. Unfortunately, optimization is largely an empirical exercise. Therefore, it is important to have robust screens to optimize the features that are most desirable. While NF-κB inhibitors (65) and aryl hydrocarbon receptor (AHR) agonists (63, 64) have been demonstrated to be tolerogenic in liposomes and on gold particles, respectively, in our hands, these immunomodulators were not active in pilot PLGA nanoparticle formulations. It is possible that further optimization would be required to create similarly active particles with PLGA. However, certain nanoparticle materials may naturally lend themselves to be more compatible with certain types of immunomodulators.

If more than one payload is needed (e.g., antigen and immunomodulator), it also important to optimize the ratio of these two components. This can be difficult as one component may affect the encapsulation efficiency of the second component, thus it may be difficult to create a series of particles in which the load of the first component is held constant while varying the load of the second component. One strategy to work around this issue is to encapsulate the two components into separate nanoparticle formulations and admix different ratios of the two particles (66). This strategy is effective if the biodistribution of the two particles is sufficiently similar. Since a therapeutic dose of nanoparticles may involve the injection of billions of nanoparticles, APCs will endocytose hundreds of nanoparticles. However, one must keep in mind that B cells specific for the target antigen may selectively take up nanoparticles containing antigen. Once an optimal ratio is determined, then a single particle formulation containing both components in the appropriate ratio can be created and tested. There is, however, a risk that the optimal ratio for mice may be different in humans. Thus, it is worth considering to develop a two particle formulation for initial human clinical trials, so that different ratios can be evaluated.

## Tolerogenic NPs That Provide Antigen Alone to Harness Natural Tolerogenic Processes or Environments

Immune tolerance is the homeostatic, default pathway for antigen presented in the absence of costimulation in steady-state, non-inflammatory environments (14, 20, 90). The immune system has evolved to distinguish between cells that undergo natural cell death, or apoptosis, versus necrotic cell death due to injury or infection. There are natural tolerogenic processes that not only maintain tolerance to self-antigens but also enable induction and maintenance of tolerance to innocuous food antigens and commensal bacterial antigens. Providing antigen in the context of these natural tolerogenic processes and environments is one strategy to induce antigen-specific tolerance.

### Peptide Antigens Presented on MHC-Bearing tNPs

T cells recognize antigenic peptides presented in the context of major histocompatibility complex (MHC) class I (CD8 T cells) or MHC class II (CD4 T cells) and require a costimulatory second signal for activation of effector T cells. In the absence of costimulation, T cells become anergic, undergo apoptosis or differentiate into regulatory T cells (12, 13). Santamaria and colleagues created “synthetic APCs” by coating iron oxide nanoparticles with specific peptide–major histocompatibility class 1 complexes (pMHC-CLI). These tNPs present antigen in the absence of costimulatory molecules resulting in suppression of autoreactive CD8+ T cells and their conversion to a regulatory, anergic phenotype that controlled pathogenic responses by a mechanism dependent on IFNγ secretion, indoleamine 2,3-dioxygenase, and perforin expression (41). Importantly, this tolerance was antigen specific and dominant as transfer of CD8+ T cells from treated mice into a naïve prediabetic NOD animal conferred protection from development of type 1 diabetes (T1D).

This approach has also been extended to MHC class II molecules loaded with disease-relevant peptides (pMHC-CLII). These tNPs were efficacious in animal models of T1D, experimental autoimmune encephalomyelitis (EAE) and arthritis, but they worked through a different mechanism than pMHC-CLI-loaded tNPs (40). Treatment with pMHC-CLII-presenting tNPs led to the differentiation of IL-10-producing Tr1 regulatory cells and regulatory B cells that were capable of transferring tolerance to untreated animals, even if the cells were antigen-experienced, suggesting that this approach could lead to reversal of memory responses.

While this approach is elegant, one of the hurdles for clinical development is the identification and validation of the relevant MHC alleles and peptides in the diverse human population. T1D and celiac disease may be the most amenable diseases for this approach, as there are strong disease associations with MHC-DQ/DR alleles. Other diseases may require GMP manufacturing of multiple tNPs bearing different MHC alleles to treat a heterogenous population.

### Harnessing Oral Tolerance

Delivery of antigen through the oral route has been shown to be tolerogenic, presumably through a mechanism that is similar to the tolerogenic response observed in all healthy individuals to dietary and gut flora antigens. However, current approaches to induce tolerance orally require chronic and frequent treatments (91). Kim et al. were among the first to load nanoparticles with antigen alone in the context of a model of collagen-induced arthritis (CIA). The authors showed that PLGA particles loaded with type II collagen (CII) provided orally 14 days before immunization with CII prevented CIA, antigen-induced T cell proliferation, anti-CIIA antibodies in a dose-dependent manner. The Peyer’s patches of treated animals had an increased TGFβ/TNFα ratio, suggesting an active anti-inflammatory program in response to the antigen (43). Dhadwar et al. showed that repeated weekly administrations of chitosan nanoparticles containing DNA encoding coagulation factor VIII (FVIII) provides sustainable FVIII activity in hemophilia A mice while avoiding the induction of inhibitory and non-neutralizing anti-FVIII antibodies (44). Goldmann et al. investigated a similar approach with ovalbumin (OVA)-encoding DNA encapsulated in chitosan tNP and showed suppressed OVA-specific delayed-type hypersensitivity (DTH) and anti-OVA antibody responses and transferable tolerance mediated by CD4+ CD25+ T cells (45).

While oral tolerance has been shown to be effective in mice, human clinical trials of oral tolerance have been largely disappointing. It is also not clear if non-viral oral delivery of DNA-bearing nanoparticles in humans would result in sufficient and sustained levels of antigen expression to induce immune tolerance.

### Harnessing Apoptotic Cell Death

Cells that undergo necrotic cell death, due to tissue damage or infection, induce an immunogenic response, while cells that undergo apoptosis, or programmed cell death, generally induce a tolerogenic response (92, 93). Coupling antigens to spleen cells through ethylenecarbodiimide (ECDI) fixation has been shown to induce their apoptosis and treatments with these dead cell-peptide conjugates confers tolerance to the antigen in many preclinical models. This approach has been used to treat various diseases, including animal models of EAE and T1D (94, 95). A similar strategy has been employed to target disease-relevant antigens to erythrocytes *in vivo*. Erythrocyte cell death led to immunologic tolerance to the bound antigen and protection in a model of autoimmune T1D (96). The adaptation of this approach to nanoparticles has involved delivery of peptide antigens coupled to dendrimers and to polystyrene and PLGA carriers. Treatment with pathogenic peptides conjugated to dendrimers protected animals from developing EAE with a concomitant reduction of effector T cells in the CNS (46). A similar approach using 500 nm carboxylated polystyrene beads with a mixture of immunodominant HLA-A*02:01-restricted epitopes was used to treat HHD II mice (β2mKO/HLA-A*0201 transgenic mouse) and inhibit diabetogenic human cytotoxic T cell (CTL) responses in a Treg-dependent manner (52). Negatively charged 500 nm PLGA nanoparticles delivered intravenously target splenic macrophages that express the scavenger receptor MARCO (47). This approach has shown to be efficacious in preventing and treating autoimmune processes in relapsing–remitting EAE and T1D and in preventing graft rejection in bone marrow transplantation and allogenic pancreatic islet transplantation (47–49, 51, 80, 81, 94, 95). Tolerance induction was demonstrated by challenging treated animals with the antigen after disease resolution or the prolonged survival of grafts (47, 48, 51) and the elimination of pathogenic effector T cells by induction of apoptosis and anergy (94, 95). The precise mechanism for the induction of tolerance by negatively charged tNPs remains to be elucidated. Recently Kuo et al. described that pro-inflammatory transcription factors NF-κB and STAT1 are triggered in macrophages and DCs when incubated with this type of nanoparticle. However, the cells had a decreased capacity for presenting antigen, displayed a restricted costimulatory-molecule phenotype with low expression of CD86, CD80, and CD40 and showed upregulation of STAT3, IL-10, and sustained PD-L1 expression, a profile associated with anti-inflammatory functions (80). Interestingly this phenotype was observed in both macrophages and DCs, while tolerance induced by negatively charged tNPs has been described to be dependent on MARCO+ macrophages only (47). It is not clear how negatively charged nanoparticles mimic apoptotic cells, or whether they recapitulate the full tolerogenic phenotype of apoptotic cells. It appears that the use of charged nanoparticles to induce tolerance works best with peptide antigens, while ECDI-fixed splenocytes and erythrocytes undergoing cell death can confer tolerance to either peptide or protein antigens. It is possible that the inherently immunogenic properties of protein antigen displayed in a multimeric fashion on NPs cannot be overcome with this approach. Interestingly, the survival of allogenic pancreatic islets graft in mice treated with poly(lactide-*co*-glycolide) particles containing alloantigen was synergistic with low dose free rapamycin, suggesting that this approach could be substantially improved by the addition of an immunosuppressant (49).

Another approach to mimic apoptotic cells is to cloak liposomes with Phosphatidylserine (PS). PS is a phospholipid forming part of the cell membrane that is translocated from the cytosolic (inner) to the extracellular (outer) membrane of cells undergoing apoptosis. Macrophages express various PS-specific scavenger receptors, such as Tyro3, Axl, and Mertk (collectively referred to as TAM receptors), TIM-3 and SCARF-1 that trigger the phagocytosis of dying cells and promote induction of a tolerogenic phenotype, such as the increase in IL-10 and TGFβ secretion, and a decrease in NF-κB signaling and TNFα, IL-1β, and IL-12 secretion (97). Encapsulating coagulation FVIII in PS-bearing liposomes was effective to prevent the formation of inhibitory anti-FVIII antibodies in hemophilia A animals even when animals were challenged with FVIII alone (53, 57, 98, 99). A similar approach was demonstrated with alpha-glucosidase (GAA) in a mouse model of Pompe disease. Administration of GAA-containing PS liposomes provided therapeutically active enzyme while preventing the formation of inhibitory antibodies (56). In an autoimmune setting, PS liposomes loaded with disease-relevant peptides were protective in the NOD animal model of T1D (54) and PLGA nanoparticles displaying PS and containing peptide autoantigens (from myelin oligodendrocyte protein, MOG_35–55_) were also efficacious in an acute model of EAE (in B6 mice) (55).

### Harnessing the Tolerogenic Environment of the Liver

The liver is considered a tolerogenic organ due to its unique function in filtering antigens from blood delivered from the gastrointestinal tract *via* the hepatic portal veins (100, 101). The liver is constantly bathed in food antigens and commensal bacterial products, “foreign” products to which immunological tolerance must be induced and maintained in healthy organisms. Carambia et al. (50) have shown that peptide-coupled poly(maleic anhydride-alt-1-octadecene)-coated nanoparticles injected i.v. protected mice in a model of EAE in a Treg-dependent manner. Microscopy studies showed selective uptake of these nanoparticles by LSECs.

## Tolerogenic NPs That Provide Antigen While Targeting Tolerogenic Receptors

One of the potential concerns about delivering nanoparticles containing only antigen is that in an inflammatory microenvironment, these tNPs could inadvertently provoke a stimulatory immune response and exacerbate an autoimmune condition. One strategy to mitigate this risk is to create tNPs that deliver antigen while simultaneously targeting tolerogenic receptors.

### Harnessing Cytokine Mediators of Immunological Tolerance

One strategy is to create a tolerogenic environment by co-delivery of antigen with nanoparticle-encapsulated anti-inflammatory cytokines and soluble mediators. Encapsulated MOG_35–55_ and rIL-10 ameliorated the course of EAE induced with MOG_35–55_ in C57BL/6 mice (58). Furthermore, two groups developed a system of multiple microparticles with different functionalities engineered to be phagocytosed and release their cargo in the intracellular space or avoid phagocytosis and release anti-inflammatory cytokines in their environment. This dual system allowed for the release of antigen and vitamin D3 inside APCs and TGFβ and GM-CSF extracellularly. Although tolerance induction was not demonstrated, treatments with these particles showed the immunoregulatory capacity of encapsulated cytokines by preventing T1D in NOD animals (59) and EAE in MOG_35–55_–immunized animals (60) consistent with a general suppressed phenotype of CD4+ T cells and a tolerogenic phenotypes on APCs. A hurdle for clinical development is the requirement to produce one or more GMP cytokines.

### Harnessing AHR Agonists

The AHR is a ligand-activated transcription factor that controls the differentiation of Foxp3+ and IL-10+ Tregs and Th17 cells. Quintana and colleagues have shown that an endogenous AHR ligand, 2-(1′H-indole-3′-carbonyl)-thiazole-4-carboxylic acid methyl ester (ITE), co-delivered with myelin peptide MOG_35–55_-loaded on gold nanoparticles promote the generation of Tregs *in vitro* and *in vivo* that were capable of transferring tolerance to naïve animals (63). This approach was also efficacious with protein antigen, as ITE and proinsulin-loaded tNPs suppressed autoimmune diabetes in NOD animals. Interestingly, treatment with these particles induced a Socs2-dependent itDC phenotype characterized the inhibition of NF-κB signaling, a decreased ability to active Teff cells and an increased differentiation of Foxp3+ Treg cells (64).

### Targeting the Fas Receptor to Kill Antigen-Specific Effector Cells

The Fas receptor mediates programmed cell death. Shen et al. created artificial APCs with latex beads coated with pMHC-CLI complexes and a monoclonal antibody directed against Fas receptor, which caused the deletion of antigen-specific CTLs in an animal model of skin graft (42). A potential hurdle for clinical development is the cost of producing GMP manufactured monoclonal antibodies.

### Targeting B Cell-Specific Tolerance through CD22

B cells play a unique role in the immune system by serving as both APCs and antigen-specific effector cells. The BCR, a transmembrane splice variant of an antibody, can directly bind to its cognate antigen and trigger B cell activation. To prevent activation to autoantigens, B cells express inhibitory co-receptors, such as CD22, a member of the SIGLEC family of lectins that binds to sialic acid-bearing glycoproteins and glycolipids. Co-localization of CD22 with the BCR results in the recruitment of phosphatases that inhibit BCR signaling and result in B cell deletion. The Paulson group developed liposomal nanoparticles, called SIGLEC-engaging tolerance-inducing antigenic liposomes (STALs) displaying both antigen and CD22 glycan ligands on their surface that induce apoptosis in mouse and human B cells. Animals treated with STALs did not develop antibody responses to T cell-independent antigens, such as nitrophenol, or T cell-dependent protein antigens, such as coagulation FVIII, even after repeated immunogenic challenges (61, 62). It is notable that this approach is compatible with the use of protein antigens, as the inhibitory signal delivered by CD22 co-engagement is sufficient to override the inherent immunogenicity of a protein-bearing nanocarrier. One potential hurdle for clinical development is the difficulty and cost of synthesizing CD22 glycan ligands.

## Tolerogenic NPs That Harness Tolerogenic Pharmacological Agents

Recently, a number of researchers have investigated pharmacological agents capable of inducing tolerogenic DCs (102–104). The potential advantage of pharmacological mediators of tolerance is the potential ability to “lock-in” a tolerogenic phenotype even in the face of an inflammatory microenvironment. While autologous tolerogenic DCs induced *ex vivo* could be used therapeutically, such individualized cell therapy would be costly and difficult to scale. However, nanoparticles represent an ideal “off-the-shelf” vehicle to deliver a payload of both target antigen and tolerogenic drug to induce endogenous tolerogenic DCs *in vivo*. To date, pharmacological agents targeting at least two different signaling pathways have been used in tNPs to induce antigen-specific tolerance *in vivo*.

### NF-κB Inhibitors

NF kappa B is a master regulator of a broad array of genes controlling inflammation and cell survival. Thomas and colleagues have demonstrated that co-delivery of antigen with various NF-κB inhibitors, such as curcumin, quercetin, and Bay11-07082, in liposomes suppressed inflammatory arthritis in an antigen-specific manner (65). The liposomes accumulated in lymph nodes and spleen following i.v. injection and were taken up by MHC class II+ APCs resulting in inhibition of NF-κB activation. Mice treated with liposomes showed induction of Ag-specific Foxp3+ regulatory T cells, which conferred protection when adoptively transferred into naïve animals. Depletion of Tregs with anti-CD25 antibodies abrogated the tolerogenic activity of the tNPs.

### mTOR Inhibitors

Mammalian target of rapamycin is a conserved serine/threonine kinase that integrates environmental signals to regulate cell metabolism and survival. Rapamycin is a natural product derived from *Streptomyces hygroscopicus*, which binds to the FK506-binding protein to form a complex that acts as an allosteric inhibitor of the mTOR complex-1 pathway. Rapamycin was found to have potent immunosuppressive activity based on its ability to inhibit T cell proliferation and is approved for the prophylaxis of renal allograft rejection. Importantly rapamycin treatment has been shown to promote Treg expansion and differentiation (27, 105, 106). In addition to its direct effects on T cells, Thomson and colleagues have demonstrated that *in vitro* treatment of DC induces a tolerogenic phenotype (25, 27).

We screened a large number of immunomodulators for compatibility with biodegradable PLA and PLGA nanoparticles and found that rapamycin-loaded nanoparticles showed potent tolerogenic activity *in vivo*. We and others have shown that tNPs containing rapamycin induced durable antigen-specific immune tolerance when coadministered with various encapsulated or free protein and peptide antigens. These tNPs were selectively taken up by APCs in lymphoid organs (66) and demonstrated efficacy when coadministered with antigen by i.v., s.c., or direct intranodal injection (66, 69). The tNPs were shown to generated itDCs and Foxp3+CD4+ T cells *in vivo* and inhibit CD4+ and CD8+ T cell effector cell activation (30, 66). Moreover, weekly doses of tNPs encapsulating rapamycin, but not daily doses of free rapamycin, were effective in inducing immune tolerance (30). Indeed, a single dose of tNPs co-encapsulating rapamycin and antigen inhibited antigen-specific T cell expansion while increasing the proportion of Foxp3+ T cells, while the equivalent doses of encapsulated antigen with free rapamycin had the opposite effect (66). This difference may be attributed to the selective targeting of tNPs to APCs in the draining lymph nodes. Importantly, immune tolerance induced by tNPs encapsulating rapamycin was effective even when coadministered with a potent TLR7/8 agonist and was maintained in animals challenged with antigen coadministered with TLR agonists or emulsified in complete Freund’s adjuvant. Tolerogenic NPs containing co-encapsulated antigen and rapamycin were effective in preventing T cell-mediated pathologies such as DTH reactions and EAE. In addition, therapeutic treatment at the peak of disease was effective in reversing paralysis in a model of EAE (66, 69). Tolerance induced by tNPs encapsulating rapamycin could be transferred to naïve animals (107).

In addition, rapamycin-containing tNPs inhibited B cell activation and differentiation into effector cells, germinal center formation and antibody production. These rapamycin-containing tNPs were effective in preventing IgE-mediated anaphylaxis in models of allergy, IgG-mediated anaphylaxis associated with repeated intravenous challenges with antigen, and the formation of anti-drug antibodies (ADAs) to a wide range of biologic drugs. Coadministration of tNPs containing rapamycin with free biologic drugs was effective in preventing ADAs against coagulation FVIII (Advate^®^) in a model of hemophilia A (66, 67); human TNFα-blocking antibody adalimumab (Humira^®^) in a model of inflammatory arthritis (30), acid-α-glucosidase (Lumizyme^®^) in a model of Pompe disease (70), recombinant immunotoxin in a model of mesothelioma (71), adeno-associated virus gene therapy vectors (68) and pegylated uricase (pegsiticase) in uricase-deficient mice and non-human primates (30). Currently the combination of tNP-rapamycin and pegsiticase (SEL-212) is in Phase 2 clinical trials (NCT02959918) in patients with symptomatic gout and hyperuricemia (see Human Translation).

## Hybrid tNPs

Strategies that employ tNPs that harness natural tolerogenic mechanisms and those that incorporate a pharmacological mediator of tolerance may have synergistic effects. Recently Paulson and colleagues added low doses of rapamycin to their STALs liposomes that present antigen in context with a CD22 ligand (108). While CD22 ligand co-localized with antigen would directly inhibit antigen-specific B cell activation, it would not prevent T cell activation by DCs and macrophages that also take up the STALs particles. Preliminary data indicate that the addition of low dose rapamycin enhanced the tolerogenic response, presumably by mitigating antigen-specific T cell activation.

## Human Translation

Currently, the treatment of autoimmune diseases requires life-long use of general immunosuppressants or immunomodulators that may target-specific pathways (e.g., TNF-α) but are not antigen-specific. A long-standing goal for immunotherapy is the development of antigen-specific therapies that leave the rest of the immune system intact and that can arrest or even reverse disease pathology. Clinical translation of other (non-nanoparticle-based) strategies to induce antigen-specific tolerance induction has been challenging and largely disappointing. The immune system is a complex network of cells, organs, and soluble factors that must integrate multiple environmental cues to determine how to respond to a given antigen. Nanoparticles are ideal vehicles to mediate antigen-specific immune modulation, as they can be engineered to provide multiple coordinated signals to shape the immune response. For example, nanoparticles have been developed for stimulatory vaccines by incorporating antigen and TLR agonists or other pro-inflammatory adjuvants. Creating tolerogenic or “inverse” vaccines using nanoparticles that harness natural tolerogenic mechanisms or employ tolerogenic pharmacological agents is an attractive concept. The preclinical data demonstrating induction of antigen-specific tolerance using a wide variety of nanoparticle materials and strategies is compelling. However, clinical translation remains a key hurdle for the field.

It is worth considering why clinical translation in immune tolerance has been so vexing. There are several factors to consider. (1) Animals models of autoimmunity are poorly predictive of human disease. In addition to obvious differences in the immune system, genetic diversity, lifespan, and environmental factors between humans and laboratory mice, there are a number of limitations of mouse models specific to autoimmune diseases. For example, many mouse models of autoimmunity are homogenous acute models that often, as in the case of EAE, use a single immunization with a single antigen to trigger pathology while most autoimmune disorders in humans are chronic and heterogenous diseases that develop over many years. (2) Antigen uncertainty. For many human autoimmune diseases, there are usually multiple candidate antigens with epitope spreading that occurs with disease progression. Moreover, the pathogenic antigens may vary from patient to patient. This antigen selection risk is compounded for peptide-based immunotherapies, as dominant peptide epitopes may vary widely among different patients due to heterogeneity in MHC alleles and T cell receptor repertoire. (3) Requirement for therapeutic activity in established disease. Modifying a memory immune response is considerably more challenging than affecting a naïve response. For example, there are many highly effective prophylactic vaccines dating back to 1796 with Jenner’s small pox vaccine; however, there is only one approved therapeutic vaccine, Provenge^®^, a modestly effective DC vaccine for prostate cancer. In mouse models of autoimmune disease, it is difficult to study true therapeutic activity in the setting of well-established disease involving memory T cell populations. Thus, it is challenging to assess whether the failure of clinical translation of immune tolerance strategies is due to non-predictive animal models, incorrect antigen selection, or short-comings of the therapeutic strategy.

To mitigate some of these challenges in assessing the clinical efficacy of rapamycin-containing tNPs, we have chosen to focus on the mitigation of ADAs to biologic therapies (30). ADAs are a common cause for biologic treatment failure and hypersensitivity reactions. Using this strategy confers several significant advantages as (1) the animal models are simple immunization models, and the clinical proof-of-concept is relatively straightforward with a well-established and easily measured biomarker readout (ADA titers), (2) the antigen is unequivocally known, as it is the biologic drug itself, and (3) the tolerizing therapy can be administered prophylactically, as it is known when the patient receives the drug. Our lead clinical program, SEL-212, is the first immune tolerizing nanoparticle technology to reach the clinic. SEL-212 is a combination therapy consisting of rapamycin-containing tNPs coadministered with pegylated uricase, a uric acid metabolizing enzyme, for the treatment of severe, chronic gout. Preclinical studies demonstrated the ability of these tNPs to prevent the formation of ADAs in uricase-deficient hyperuricemic mice enabling the enzyme to achieve sustained control of serum uric acid levels (30). Similar effects on ADA prevention were obtained in rats and non-human primates (30). A single ascending dose Phase 1 clinical trial of SEL-212 (NCT02648269) in patients with hyperuricemia showed that the pegylated uricase is highly immunogenic in humans even after a single dose of enzyme. The addition of tNPs showed a dose-dependent inhibition of anti-uricase antibody formation resulting in sustained reduction of serum uric acid levels (72). An ongoing Phase 2 study (NCT02959918) will assess the ability of multiple doses of SEL-212 to inhibit the formation of ADAs with in patients with symptomatic gout and hyperuricemia.

## Author Contributions

RM and TK wrote and edited this review.

## Conflict of Interest Statement

Both RM and TK are employees and shareholders of Selecta Biosciences Inc., a company developing tolerogenic nanoparticles. The handling editor and reviewer HW declared their involvement as coeditors in the Research Topic, and confirm the absence of any other collaboration.
